# Predictive value of N-terminal pro-brain natriuretic peptide among critically ill patients

**DOI:** 10.1186/cc11008

**Published:** 2012-03-20

**Authors:** M Cubrilo-Turek, N Maric, I Mikacic, N Tolj Karaula, N Budinski, M Mackovic

**Affiliations:** 1 Clinical Hospital Sveti Duh, Zagreb, Croatia

## Introduction

N-terminal pro-brain natriuretic peptide (NT-proBNP) represents a useful cardiac marker in evaluating heart failure. However, its role in the assessment of critically ill patients is not clear. The aim of this study was to evaluate survival of infected and noninfected patients according to the measurements of NT-proBNP.

## Methods

Serum NT-proBNP measurements were done in 89 (46 males/43 females, 68.20 ± 13.80 years) consecutive critically ill patients within 6 hours after admission to the ICU. NT-proBNP was determined with a sandwich immunoassay on an Elecsys 2010 (Roche Diagnostics, Mannheim, Germany). Logarithmic transformation of data was required because of the skewed distribution of NT-proBNP.

## Results

The median NT-proBNP (pg/ml) was 2,485.1 pg/ml (range 31.5 to 12,041 pg/ml) (log NT-proBNP mean 3.34 ± 0.71 pg/ml). Mean log NT-proBNP levels were higher at admission to the hospital in nonsurvivors (3.73 ± 0.67 pg/ml) compared with survivors (3.12 ± 0.65 pg/ml), which was statistically significant (*P *< 0.0001). Higher concentrations were found in proven infection (X ± SD) (3.43 ± 0.68) than in bacteriological negative patients (3.30 ± 0.72), but it was statistically insignificant (*P *< 0.42). From 57 survivors seven were mechanically ventilated (12.28%) while 14 (43.75%) from 32 nonsurvivors were ventilated, which was statistically significant (*P *< 0.001). More nonsurvivors were taking vasoactive medications (*n *= 12 or 37.5%) than survivors (*n *= 3 or 5.26%), which was statistically significant (*P *< 0.001). NT-proBNP showed no correlation for any analyzed parameters (age, erythrocytes, leucocytes, body temperature, systolic and diastolic blood pressure, C-reactive protein, fibrinogen, lactates or procalcitonin). The use of ROC curve analysis reveals for serum NT-proBNP high sensitivity (75%), low specificity (57.9%) and low accuracy (64%) for discriminating survivors from nonsurvivors.

## Conclusion

Our results showed that cardiac NT-proBNP levels can be elevated in critically ill patients and may also serve as markers of severity and prognosis for survival. Mean baseline levels of log NT-proBNP were different in critically ill patients with proved bacteriological infection than in patients without proven infection.

